# Ethnic disparities in mortality among overweight or obese adults with newly diagnosed type 2 diabetes: a population-based cohort study

**DOI:** 10.1007/s40618-021-01736-9

**Published:** 2022-01-13

**Authors:** B. Iyen, Y. Vinogradova, R. K. Akyea, S. Weng, N. Qureshi, J. Kai

**Affiliations:** Primary Care Stratified Medicine, Population Health and Lifespan Sciences, University of Nottingham, Nottingham, UK

**Keywords:** Ethnicity, Incidence, Mortality, Obese, Overweight, Type 2 diabetes

## Abstract

**Purpose:**

Ethnic variation in risk of type 2 diabetes is well established, but its impact on mortality is less well understood. This study investigated the risk of all-cause and cardiovascular mortality associated with newly diagnosed type 2 diabetes in White, Asian and Black adults who were overweight or obese.

**Methods:**

This population-based cohort study used primary care records from the UK Clinical Practice Research Datalink, linked with secondary care and death registry records. A total of 193,528 obese or overweight adults (BMI of 25 or greater), with ethnicity records and no pre-existing type 2 diabetes were identified between 01 January 1995 and 20 April 2018. Multivariable Cox proportional hazards regression estimated hazards ratios (HR) for incident type 2 diabetes in different ethnic groups. Adjusted hazards ratios for all-cause and cardiovascular mortality were determined in individuals with newly diagnosed type 2 diabetes.

**Results:**

During follow-up (median 9.8 years), the overall incidence rate of type 2 diabetes (per 1,000 person-years) was 20.10 (95% CI 19.90–20.30). Compared to Whites, type 2 diabetes risk was 2.2-fold higher in Asians (HR 2.19 (2.07–2.32)) and 30% higher in Blacks (HR 1.34 (1.23–1.46)). In individuals with newly diagnosed type 2 diabetes, the rates (per 1,000 person-years) of all-cause mortality and cardiovascular mortality were 24.34 (23.73–24.92) and 4.78 (4.51–5.06), respectively. Adjusted hazards ratios for mortality were significantly lower in Asians (HR 0.70 (0.55–0.90)) and Blacks (HR 0.71 (0.51–0.98)) compared to Whites, and these differences in mortality risk were not explained by differences in severity of hyperglycaemia.

**Conclusions/Interpretation:**

Type 2 diabetes risk in overweight and obese adults is greater in Asian and Black compared to White ethnic populations, but mortality is significantly higher in the latter. Greater attention to optimising screening, disease and risk management appropriate to all communities with type 2 diabetes is needed.

**Supplementary Information:**

The online version contains supplementary material available at 10.1007/s40618-021-01736-9.

## Introduction

The global prevalence of type 2 diabetes mellitus (T2DM) has been increasing steadily for the past 3 decades [1], with 80–85 percent of this burden resulting from obesity and overweight [2]. In 2019, an estimated 463 million people were living with diabetes, and this is predicted to increase to 578 million people by 2030. There is well established ethnic variation in the risk of developing T2DM, with significantly higher risks observed in Asians and Blacks than White Europeans [3, 4]. While it is known that T2DM increases the risk of death [5, 6], there is conflicting evidence on how increased mortality risk associated with T2DM varies among ethnic populations [7, 8]. Recent work suggests previously observed excess mortality in South Asians with T2DM, may have been reversed [9]. In particular, it is not known whether the risk of mortality in overweight or obese individuals with T2DM differs by ethnicity.

Given the rising global burden of obesity and disproportionate risk of T2DM among different ethnic populations, this study aimed to explore any potential evidence of inequalities in mortality outcomes and identify factors which may be related to any differences in outcomes. We determined the incidence of T2DM in a large population-based cohort of White, Asian and Black overweight and obese adults and then investigated the risk of all-cause mortality and cardiovascular mortality following a diagnosis of T2DM.

## Methods

### Data source

The UK Clinical Practice Research Datalink (CPRD) is a large electronic database of anonymised longitudinal routine primary care health records, sourced from a network of over 1,800 general practices, and includes 50 million patients of which 14 million are currently registered and active [10, 11]. For this study, we obtained data on individuals who had records in CPRD from 01 January 1995 to 20 April 2018. Primary care records in CPRD were linked to individuals’ secondary care records (Hospital episode statistics) and death registry records from the Office of National Statistics (ONS). Data access and ethical approval for the study was granted by the CPRD Independent Scientific Advisory Committee (Protocol number 18_195A).

### Study population

The study cohort comprised individuals aged 18 years or over, who were overweight or obese (body mass index (BMI) of 25 kg/m^2^ or greater), free from T2DM at baseline, and had ethnicity documented in their records. We excluded individuals who had been registered at their general practice for less than 1 year, as a means of ensuring that those with prevalent T2DM were not included in the study. All study participants were followed-up until a diagnosis of T2DM, death, transfer out of the practice or the last date of data collection, whichever occurred first.

### Categories of ethnicity

Ethnicity was categorized differently in CPRD database and HES. In HES records, individuals were categorized into one of twelve ethnic groups—Bangladeshi, Black African, Black Caribbean, Black other, Chinese, Indian, Mixed, Other Asian, Pakistani, White or other, whereas ethnicity in CPRD was categorised into one of nine ethnic groups—Bangladeshi, Black African, Caribbean, Chinese, Indian, other Asian, Pakistani, White (UK or European) or other. We used HES as the primary source of data for ethnicity and all ethnicity records that were missing in HES were replaced with the record in CPRD when available. After extracting records of ethnicity, these were re-categorised into the broad ethnic categories used by the Office for National Statistics in the UK 2001 census [12], ensuring that categories used were standardised and generalizable. In this study, we evaluated the 3 ethnic groups that are clearly defined by the Office for National Statistics:

- Asian (includes Bangladeshi, Chinese, Indian, Pakistani, Asian Other).
- Black (includes Black African, Black Caribbean, Black other).
- White (includes White Irish, White Gypsy/Traveller, White other).

### Other covariates

Individuals’ characteristics such as age, sex, socioeconomic status (measured using index of multiple deprivation (IMD) quintiles) [13], smoking status, alcohol consumption as well as variables that could alter the risk of type 2 diabetes or death were identified from electronic health records. Other variables collected at baseline include family history of T2DM, statin prescribing, use of corticosteroids and history of comorbidities: atrial fibrillation, chronic kidney disease, hypertension, coronary heart disease, stroke, peripheral vascular disease, heart failure and rheumatoid arthritis/other inflammatory diseases [14]. In individuals with incident diagnosis of T2DM, we identified the levels of glycated haemoglobin (HbA1c) at the time of T2DM diagnosis as well as 1 year after diagnosis [15].

### Outcome ascertainment

The primary outcome was the first recorded diagnosis of type 2 diabetes identified from primary care or secondary care records. The secondary outcome was mortality among those with a diagnosis of type 2 diabetes. Disease identification in primary care used the T2DM disease codes (shown in the supplemental online file), and identification from HES secondary care records used the ICD-10 code E11. Death records were identified from individuals’ primary care record, secondary care or ONS death registry records. Cardiovascular-related deaths were identified from ONS death registry records when an underlying primary cause of death included ICD-10 codes for coronary heart disease (I20-I25), stroke or TIA (I60-I69, G45), peripheral vascular disease (I70-I73) or heart failure (I50).

### Statistical analyses

Baseline characteristics of the study population were determined for different ethnic groups and presented with missing variables. Data were expressed as proportions, mean (standard deviation) and median (interquartile range) for categorical, continuous normally distributed and continuous non-normally distributed variables, respectively. BMI was analysed as a categorical variable, using the standard WHO BMI classification categories [16]. Appropriate statistical tests such as chi-squared and analyses of variance tests (ANOVA) were used to assess differences in categorical and continuous variables, between the different ethnic groups. We determined the crude incidence rates of T2DM in different ethnic groups and then incidence rate ratios were estimated with Whites being the referent category. Using multivariable Cox proportional hazards regression analyses, we estimated the hazards ratio for T2DM in different ethnic groups. In the second phase of analyses restricted to the subgroup of individuals with new-onset T2DM, individuals were followed-up from the date of recorded T2DM diagnosis, and then the rates of all-cause mortality and cardiovascular-related mortality were determined. Multivariable Cox proportional hazards regression was used to derive the hazards ratio for mortality in different ethnic groups versus Whites. All multivariable Cox analyses were adjusted for the range of sociodemographic and co-existing clinical covariates described above. Only a small proportion of individuals had missing smoking or alcohol data (0.1% and 2.8%, respectively) and those with missing values of these covariates were assumed to be a random sample of the total population, so complete case analyses was adopted when analyses included these variables [17]. In individuals with missing or unrecorded categorical variables such as family history of T2DM or clinical comorbidities, the common assumption was made that these individuals did not have the condition and they were assigned null values for these variables. This preserved the sample size while taking account of the missing values in the analyses.

To explore whether any observed ethnic variation in risk of mortality in individuals with newly diagnosed T2DM might be explained by differences in the severity of hyperglycaemia, we determined and assessed for any differences in HbA1c characteristics of the population at the time of T2DM diagnosis and 1 year after diagnosis. We did not include data on T2DM therapy, as change in HbA1c is a primary endpoint of most T2DM treatments. Further, we included HbA1c at diagnosis into the adjusted Cox models to evaluate the impact of these on the adjusted hazards ratios for mortality.

The main analyses were conducted on all eligible study participants. To ensure that our study findings were robust, we did sensitivity analyses by repeating the analyses on only the subset of individuals with linked secondary care data. All analyses were conducted using Stata SE version 15.

## Results

A total of 193,528 overweight or obese adults in CPRD database had records of ethnicity and no pre-existing T2DM. Linked HES secondary care data were available for 139,520 (72%) of these individuals. Of the total study population, 94.5% were White Caucasians, 3.1% were Asians and 2.4% were Black. In the non-White ethnic groups, the prevalence of overweight and obesity increased with increasing levels of deprivation, with the steepest gradient observed in the Black ethnic population. Alcohol consumption and smoking were highest among Whites, and lowest among Asians, with 73.5% and 82% of Asians being non-alcohol drinkers and non-smokers, respectively. Asians had the highest proportion of individuals with a family history of T2DM (31%) compared to other ethnic groups. Medical conditions such as atrial fibrillation (AF), chronic kidney disease (CKD), coronary heart disease (CHD), stroke, peripheral vascular disease (PVD) and heart failure, were most common in Whites than in other ethnic populations. Whites also had the highest prescribing rates of statins and oral corticosteroids than other ethnic groups. Hypertension was most common in Blacks while rheumatoid arthritis was most common among Asians.

The characteristics of the study population by ethnic group are shown in Table 1.

**Table 1:** Baseline characteristics of the study population of obese/overweight individuals, by ethnic group (*n* = 193,528)

	White *N* = 182,887 (94.50%)	Asian *N* = 6,061 (3.13%)	Black *N* = 4,580 (2.37%)	*p* value‡
Age in years (mean (SD))	50.6 (13.1)	45.5 (11.4)	45.3 (10.4)	< 0.0001
Female *n* (%)	112,014 (61.3)	3896 (64.3)	3483 (76.1)	< 0.0001
Body mass index (kg/m^2^) ((mean (SD)	33.5 (5.9)	32.0 (5.0)	34.3 (5.9)	< 0.0001
BMI categories (kg/m^2^)				< 0.0001
Overweight (25.0–29.9)	56,104 (30.7)	2349 (38.8)	1111 (24.3)	
Obesity class 1 (30–34.9)	66,451 (36.3)	2422 (40.0)	1747 (38.1)	
Obesity class 2 (35–39.9)	36,454 (19.9)	886 (14.6)	1019 (22.3)	
Obesity class 3 (> 40)	23,878 (13.1)	404 (6.7)	703 (15.4)	
Deprivation quintile *n* (%)				< 0.0001
1 (least deprived)	24,095 (13.2)	497 (8.2)	152 (3.3)	
2	28,897 (15.8)	762 (12.6)	265 (5.8)	
3	26,447 (14.5)	926 (15.3)	526 (11.5)	
4	27,252 (14.9)	1217 (20.1)	1243 (27.1)	
5 (most deprived)	22,281 (12.2)	1196 (19.7)	1371 (29.9)	
Missing records	53,915 (29.5)	1463 (24.1)	1023 (22.3)	
Smoking status *n* (%)				
Non-smoker	91,588 (50.1)	4969 (82.0)	3547 (77.5)	< 0.0001
Ex-smoker	60,123 (32.9)	586 (9.7)	588 (12.8)	
Light smoker (1–9 cigs/day)	12,983 (7.1)	302 (5.0)	270 (5.9)	
Moderate smoker (10–19/day)	9788 (5.4)	135 (2.2)	120 (2.6)	
Heavy smoker (20–39/day)	8204 (4.5)	63 (1.0)	55 (1.2)	
Missing	201 (0.1)	6 (0.1)	0 (0.0)	
Alcohol consumption *n* (%)				< 0.0001
No alcohol consumption	53,689 (29.4)	4458 (73.5)	2506 (54.7)	
Trivial (< 1 unit/day)	63,311 (34.6)	890 (14.7)	1390 (30.4)	
Light (1–2 units/day)	29,678 (16.2)	281 (4.6)	343 (7.5)	
Moderate (3–6 units/day)	15,194 (8.3)	101 (1.7)	70 (1.5)	
Heavy (7–9 units/day)	5321 (2.9)	37 (0.6)	31 (0.7)	
Very heavy (> 9 units/day)	3310 (1.8)	25 (0.4)	23 (0.5)	
Ex alcohol drinker	7247 (4.0)	86 (1.4)	90 (2.0)	
Missing	5137 (2.8)	183 (3.0)	127 (2.8)	
Family history of T2DM *n* (%)	26,023 (14.2)	1876 (31.0)	1066 (23.3)	< 0.0001
Comorbidities *n* (%)				
Atrial fibrillation	11,061 (6.1)	80 (1.3)	61 (1.3)	< 0.0001
Chronic kidney disease	18,734 (10.5)	269 (4.5)	271 (6.0)	< 0.0001
Hypertension	71,214 (39.7)	1932 (32.9)	1855 (41.2)	< 0.0001
Coronary heart disease	25,683 (14.2)	543 (9.2)	171 (3.8)	< 0.0001
Stroke	8361 (4.6)	126 (2.1)	100 (2.2)	< 0.0001
Peripheral vascular disease	3966 (2.2)	36 (0.6)	28 (0.6)	< 0.0001
Heart failure	7443 (4.1)	123 (2.0)	91 (2.0)	< 0.0001
Rheumatoid arthritis	8196 (4.5)	301 (5.0)	133 (2.9)	< 0.0001
Prescribed medication *n* (%)				
Statins	71,575 (42.6)	1801 (34.0)	929 (21.9)	< 0.0001
Corticosteroid use	53,338 (29.8)	1631 (27.7)	734 (16.2)	< 0.0001

^‡^ANOVA test was used for comparison between continuous variables with normal distribution, and test of significance for categorical variables were derived using the Pearson’s χ^2^ test

### Incidence of type 2 diabetes

During a median follow-up of 9.8 years (IQR 5.9–13.5) [1,924,200 person-years], a total of 38,166 incident cases of T2DM were identified. The overall incidence rate of T2DM (per 1000 person-years) was 20.10 (95% CI 19.90–20.30). Variation in T2DM incidence rates among obese and overweight individuals were observed between ethnicities (Table 2). Individuals of Asian ethnicity were younger at time of T2DM diagnosis and they had the highest incidence rate of T2DM, per 1,000 person-years: 35.33 (95% CI 33.69–37.06), followed by those of Black ethnicity: 21.20 (19.77–22.74), and the lowest prevalence was observed in the White ethnic group: 19.67 (19.47–19.88). Compared to White overweight and obese adults, the incidence rate ratio for type 2 diabetes in Black overweight and obese adults was 1.08 (95% CI 1.01–1.16) and the rate ratio in Asians was 1.80 (1.71–1.89).

**Table 2:** Incidence of type 2 diabetes in adults who are overweight or obese (n = 193, 528)

	White *N* = 182,887 (94.50%)	Asian *N* = 6,061 (3.13%)	Black *N* = 4,580 (2.37%)
New-onset diabetes *n* (%) *n* = 38,166	35,692 (19.5)	1689 (27.9)	785 (17.1)
Age at T2DM diagnosis in years (mean (SD))	56.7 (11.6)	52.6 (11.3)	54.7 (11.3)
Follow-up (1000 person-years)	1814.2	47.8	37.0
Incidence rate (95% CI) of T2DM/1000 p-yrs	19.7 (19.5–19.9)	35.3 (33.7–37.1)	21.2 (19.8–22.7)
Incidence rate ratio (baseline = white)	1.0	1.8 (1.7–1.9)	1.1 (1.0–1.2)
Incidence rate of T2DM by BMI categories (kg/m^2^) /1000 p-yrs			
Overweight (25.0–29.9)	10.9 (10.7–11.2)	28.5 (26.3–30.9)	15.9 (13.6–18.5)
Obesity class 1 (30–34.9)	19.7 (19.3–20.0)	37.7 (35.0–40.6)	21.4 (19.1–23.9)
Obesity class 2 (35–39.9)	27.1 (26.5–27.6)	43.0 (38.3–48.3)	21.6 (18.5–25.1)
Obesity class 3 (> 40)	34.8 (34.0–35.7)	50.2 (42.6–59.2)	31.7 (27.0–37.1)

**Fig. 1:**
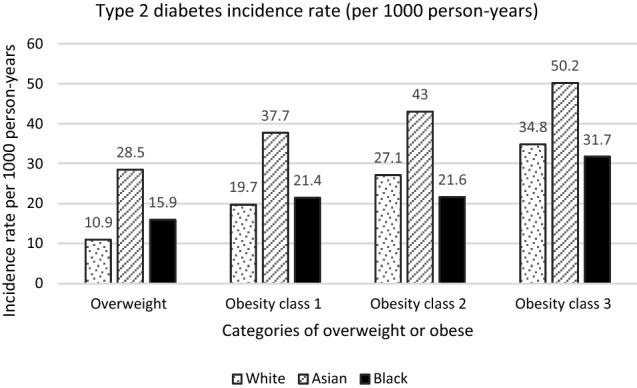
Incidence of type 2 diabetes in overweight and obese adults of White, Asian and Black ethnic groups

In keeping with expected trends, T2DM incidence rates in all ethnic populations were observed to increase with increasing levels of obesity (Fig. 1). We, however, noted that across all BMI categories of overweight or obesity, Asians had significantly higher incidence rates of T2DM than other ethnic populations. In the overweight and obese class 1 categories, Blacks had higher incidence rates of T2DM than Whites, but this was not significant in the class 1 obese group. Conversely, among those with class 2 obesity, Whites had significantly higher incidence rates of T2DM than Blacks. Higher incidence rate of T2DM was also noted in Whites compared with Blacks with class 3 obesity, but this was not statistically significant.

Table 3 shows the hazards ratios for T2DM in Asian and Black compared to White ethnic groups. After adjusting for age, sex, baseline BMI, smoking, alcohol, socioeconomic deprivation, family history of T2DM and clinical comorbidities, Black adults were over 30% more likely to have incident T2DM while Asians had a twofold higher risk of T2DM than White adults who were overweight or obese.

**Table 3:** Risk of type 2 diabetes in obese or overweight Blacks and Asians, compared to those of White ethnicity

Ethnic group	Unadjusted hazards ratio (95% CI)	Adjusted hazards ratio (95% CI)		
		Model adjusted for age and sex	Model adjusted for baseline sociodemographic characteristics^a^	Model adjusted for baseline sociodemographic characteristics and clinical risk factors Ω
White	1.00	1.00	1.00	1.00
Asian	1.79 (1.70–1.88)	2.08 (1.98–2.18)	2.49 (2.35–2.64)	2.19 (2.07–2.32)
Black	1.07 (1.00–1.15)	1.33 (1.23–1.42)	1.23 (1.13–1.34)	1.34 (1.23–1.46)

Ω Adjusted for age, sex, BMI, smoking, alcohol, deprivation, hypertension, family history of T2DM, corticosteroid use and statin use

^a^Adjusted for age, sex, BMI, smoking, alcohol and deprivation

### Mortality among newly diagnosed overweight and obese type 2 diabetic individuals

The 38,166 overweight or obese adults with newly diagnosed T2DM were followed-up for a median of 5.75 years (IQR 2.80–9.28) from the date of T2DM diagnosis. A total of 5,865 deaths (15.37%) occurred during this period, with 1,151 being CVD-related deaths (3% of T2DM population) (Table 4). Overall, the all-cause mortality rate (95% CI) per 1,000 person-years was 6.66 (6.50–6.84), and CVD mortality rate (95% CI) per 1,000 was 1.31 (1.24–1.39).

**Table 4:** Mortality rates among obese or overweight adults with new-onset diabetes (*n* = 38,166)

	White *N* = 35,692	Asian *N* = 1,689	Black *N* = 785
All-cause mortality			
Deaths among incident T2DM subjects	5,733 (16.06)	82 (4.85)	50 (6.37)
All-cause mortality rate ((95% CI) per 100,000 p-yrs	6.92 (6.75–7.11)	2.26 (1.82–2.80)	3.17 (2.40–4.18)
All-cause mortality, by BMI			
Overweight (25.0–29.9)	8.68 (8.22–9.17)	2.33 (1.61–3.37)	4.13 (2.40–7.12)
Obesity class 1 (30–34.9)	7.01 (6.72–7.32)	1.78 (1.22–2.60)	2.82 (1.78–4.47)
Obesity class 2 (35–39.9)	6.18 (5.86–6.53)	2.53 (1.53–4.20)	2.50 (1.25–4.99)
Obesity class 3 (> 40)	6.10 (5.73–6.49)	3.69 (2.09–6.49)	3.63 (2.01–6.56)
CVD-related mortality			
Number of CVD deaths	1125	18	8
CVD mortality rate (95% CI) per 100,000 p-yrs	1.36 (1.28–1.44)	0.50 (0.31–0.79)	0.51 (0.25–1.01)

All-cause mortality and CVD mortality rates were substantially lower in Asians and Blacks than in Whites (Table 4). All-cause mortality rate in Whites was 6.92 (6.75–7.11) per 1,000 person-years, mortality rate in Blacks was 3.17 (2.40–4.18) and the rate in Asians was 2.26 (1.82–2.80).

Table 5 shows that the hazards ratio for all-cause mortality in overweight, class 1 and class 2 obese individuals with newly diagnosed T2DM was significantly lower in Asians and Blacks compared to Whites. In those with class 3 obesity, the hazards ratio for mortality was non-significantly lower in Asians and Blacks compared to Whites.

**Table 5:** Hazards ratios for all-cause mortality in overweight and obese Black and Asian newly diagnosed diabetic individuals compared to Whites

	White *N* = 35,692	Asian *N* = 1,689	Black *N* = 785
Overweight (BMI 25.0–29.9)			
Unadjusted hazards ratio (95% CI)	1.00	0.27 (0.19–0.39)	0.49 (0.28–0.84)
Adjusted hazards ratio ¥	1.00	0.62 (0.40–0.97)	0.64 (0.33–1.24)
Obesity class 1 (BMI 30–34.9)			
Unadjusted hazards ratio (95% CI)	1.00	0.26 (0.18–0.38)	0.40 (0.25–0.64)
Adjusted hazards ratio ¥	1.00	0.61 (0.40–0.95)	0.77 (0.44–1.33)
Obesity class 2 (BMI 35–39.9)			
Unadjusted hazards ratio (95% CI)	1.00	0.44 (0.26–0.73)	0.43 (0.21–0.85)
Adjusted hazards ratio ¥	1.00	0.78 (0.45–1.36)	0.64 (0.26–1.55)
Obesity class 3 (BMI > 40)			
Unadjusted hazards ratio (95% CI)	1.00	0.61 (0.35–1.08)	0.66 (0.36–1.19)
Adjusted hazards ratio ¥	1.00	0.87 (0.49–1.55)	0.73 (0.38–1.42)

¥ Adjusted for age of T2DM diagnosis, sex, baseline BMI, alcohol, smoking, deprivation, coronary heart disease, stroke, peripheral vascular disease, heart failure and statin use

On adjustment for baseline sociodemographic characteristics, clinical comorbidities and statin use, there remained a statistically significant lower risk of mortality in Asians compared to Whites with BMI in the overweight and obese class 1 category. In more severely obese BMI categories (obese class 2 and 3), the lower mortality risk observed in Asians compared to Whites was not statistically significant in adjusted analyses. There was also no significant difference in adjusted hazards ratio for mortality between Whites and Blacks of all categories of overweight or obesity. In addition, no statistically significant difference was observed in the unadjusted and adjusted risk of mortality between Asian and Black ethnic individuals with newly diagnosed T2DM, irrespective of the class of obesity or overweight.

On assessing HbA1c in the different ethnic groups, Asians and Blacks had higher levels of HbA1c than Whites, at time of T2DM diagnosis. In all ethnic groups, there were decreases in HbA1c levels 1 year after T2DM diagnosis, however, these levels remained higher in individuals of Asian and Black than in White ethnic groups (Supplemental table A). On including HbA1c at time of T2DM diagnosis into the multivariable Cox models, the risk of mortality in newly diagnosed T2DM remained significantly lower in Asians than Whites (HR 0.57 (0.42–0.79)) in the overweight and obese class 1 categories only, but no significant difference in mortality risk between Whites and Asians with more severe forms of obesity. Blacks had non-significantly lower mortality risks than Whites across all categories of obesity and overweight (Supplemental table B).

### Sensitivity analyses

On restricting all the analyses to only the subset of individuals with linked secondary care records, the hazards ratios for incident T2DM remained significantly higher in Asians and Blacks compared with Whites although there was a slight attenuation of the estimates (Supplemental table C). In individuals with newly diagnosed T2DM and linked secondary care records, the hazards ratios for mortality in Asian and Black compared with White groups, remained unchanged from estimates derived from the entire cohort (Supplemental table D).

Lastly, mortality rates and hazards ratios were assessed in the population of individuals who did not develop T2DM during follow-up. In this analysis, the unadjusted hazards ratio for mortality was higher in Whites compared to Asian and Black ethnic groups. However, on adjusting for sociodemographic, lifestyle factors and clinical comorbidities, no significant difference was observed in mortality rate between the overweight and obese non-diabetic population of adults from different ethnic groups (Supplemental tables E and F).

## Discussion

### Statement of principal findings

This large cohort study of overweight and obese adults has found ethnic disparities in mortality associated with newly diagnosed T2DM, while confirming disparities in risk of T2DM. Risk of T2DM incidence was significantly higher in Asian and Black compared to White ethnic groups. However, those diagnosed with T2DM from Asian and Black groups experienced lower all-cause and cardiovascular mortality than those from White ethnic groups. After adjusting for sociodemographic, lifestyle and clinical comorbidities, there remained a statistically significantly higher risk of mortality in overweight and obese class 1 T2DM Whites compared to Asians but no statistical difference in mortality between Whites and Asians with more severe forms of obesity, as well as between Whites and Blacks with T2DM. Adjusting for level of glycaemia at diagnosis did not alter the significantly higher mortality risk in overweight and obese Whites compared to Asians with T2DM.

### Strengths and weaknesses of the study

To our knowledge, this is the first study to investigate ethnic differences in the risk of all-cause and cardiovascular mortality associated with newly diagnosed T2DM, among overweight and obese adults. Strengths include the large sample size, extensive follow-up and high-quality data source [11] enhancing generalisability of our findings to the general population of individuals who are overweight or obese. Linkage of primary care electronic health records with secondary care records enabled comprehensive ascertainment of T2DM and mortality outcomes. We systematically analysed T2DM incidence and mortality outcomes using consistent methodology to adjust for sociodemographic and comorbid confounders and did sensitivity analyses to ensure that our study findings were robust. Applying methodological principles of causal inference, we investigated whether the association between T2DM and mortality risk in the different ethnic groups was modified by severity of hyperglycaemia (HbA1c), and this is a major strength and novel feature of our study.

We recognise study limitations. These include lack of recorded data on physical activity, diet, type of diabetic treatment and blood pressure-lowering treatment, which could potentially influence T2DM and its associated outcomes [18–20]. In particular we were limited to using the UK Office for National Statistics 2001 census’ broad categories for ethnicity [12] which under-estimates the sociocultural diversity within these categories. These categories have, however, enabled identification of practical ethnic groupings large enough for meaningful statistical analyses. The study population was predominantly white, reflecting the ethnic distribution of the UK general population, but we recognise the proportion of Asians and Blacks in our population was modest and lower than estimates reported in the 2011 UK census data [12]. Our study used records available in electronic GP and hospital databases, and the ethnic disproportion presented is similar to previous database studies [14] and other studies on ethnicity [3].

Whilst indices such as waist circumference and waist-hip ratio measure degree of body fat distribution more accurately than BMI, we have used BMI data available as the most widely used measure in clinical practice. The BMI measures in the study were from healthcare professionals’ entries and so, are likely to be more accurate than self-reported measures. Although evidence has shown that BMI thresholds for disease risk is lower in Asians, this study used the standard definition of obesity and overweight routinely used in clinical practice within the UK. Our findings, therefore, provide pragmatic estimates of T2DM and mortality risks as defined by clinicians and based on routine clinical measures. The use of Index of Multiple Deprivation (IMD) as proxy for deprivation does have some limitations, such as being an area-based relative measure, rather than an individualised measure of deprivation. Lastly, overweight and obesity were determined using BMI at baseline and we did not take account of changes in BMI over time. We know, however, from previous analyses of BMI trajectory in this study population, and in other obese populations, that BMI of obese individuals does not change significantly over time [21–23].

### Comparison with previous literature

Although previous studies have explored the risks of T2DM incidence among different ethnic populations [3, 4, 24], this study uniquely explored the risk of T2DM associated with ethnicity in a population of individuals who were overweight or obesity. We included only individuals with BMI of 25 kg/m^2^ and above due to the possibility that lower BMI subjects who develop T2DM may have a stronger genetic predisposition to T2DM [25], more severe forms of disease and worse prognosis.

The disproportionately higher risk of T2DM in Asians compared to Whites and Blacks in our study persisted even after adjustment for baseline sociodemographic characteristics and clinical risk factors. This provides confirmatory and contemporary evidence of ethnic variation in risk of T2DM and that this disparity persists despite it being noted over two decades earlier [26]. While hereditary predisposition including difference in central fat distribution and the resulting insulin resistance in some ethnic groups may play a part [27], more remedial environmental, lifestyle and developmental factors have also been implicated [28].

The increased risk of all-cause and cardiovascular mortality associated with T2DM is well established [5, 6] but only a small number of studies have evaluated these risks in relation to ethnicity. Unlike previous studies, ours is the first to assess severity of hyperglycaemia at time of T2DM diagnosis and explore whether this modified the association between T2DM and mortality risk among the ethnic groups. We observed lower all-cause and cardiovascular mortality among individuals with newly diagnosed T2DM from Asian and Black groups compared those from the White group. Although this higher mortality risk in Whites with newly diagnosed T2DM was largely explained by, and disappeared after adjusting for, confounders such as sociodemographic, lifestyle and clinical comorbidities, there remained a significantly higher risk of mortality in overweight and obese class 1 Whites compared to Asians. Similar to our study, two population-based Canadian cohort studies [24, 29] found substantially lower mortality in South Asians and Chinese individuals with newly diagnosed T2DM than in White individuals. T2DM was also associated with more years of life lost among White compared to South Asian or Black individuals in a cohort study using electronic health records in the UK [7]. This emerging evidence contrasts with much earlier research from almost 3 decades ago which suggested higher mortality in Asian and Black than White individuals with T2DM, but was limited by the use of place of birth as proxy for ethnicity, and T2DM diagnosis assigned when an individual’s death certificate listed T2DM as a cause of death [8].

Lastly, while higher mortality in the obese is well established [30, 31], including in ethnic groups [31, 32], the current study is the first to explore mortality risks in an ethnically diverse population of overweight or obese individuals with newly diagnosed T2DM and thus newly adds to existing research.

### Implications for practice and research

Clinical comorbidities and cardiovascular risk factors such as smoking, and alcohol consumption were more prevalent in White than Asian and Black ethnic groups, and the higher risk of mortality observed in Whites compared with Blacks with T2DM, as well as in Whites compared with Asians with more severe forms of obesity, appeared to be largely explained by sociodemographic, lifestyle as well as clinical comorbidities. We do not have clear explanations for the observed higher risk of mortality in overweight and obese class 1 adults with T2DM from White compared to Asian groups. Although there was robust adjustment for confounders in our analyses, we cannot rule out the possibility that the observed differential mortality risk in our ethnic populations, might be partly due to other unmeasured confounders.

Earlier T2DM diagnosis with potentially earlier T2DM management and commencement of cardiovascular risk-reducing agents have been postulated as possible mechanisms underlying a shift in the pattern of cardiovascular mortality over time, in Asians with T2DM [9]. T2DM was diagnosed at an earlier age in Asian and Black compared to White ethnic groups in our study. This may be due to improved clinical and public awareness with earlier T2DM diagnosis in these communities, including lower BMI thresholds for risk assessment advanced for Asians in 2002 [33]. However, as Asian and Black individuals in our study had worse glycaemic levels than White individuals, both at the time of T2DM diagnosis and 1 year after diagnosis, there is no evidence to suggest more aggressive T2DM management in these ethnic groups after diagnosis. Further evidence is, therefore, needed to understand the mechanism underlying the differential mortality risk associated with T2DM in ethnic populations.

## Conclusion

This study has found that in the general population of adults who are overweight or obese, the risk of T2DM is highest in Asian followed by Black and then White ethnic groups. Compared to White individuals, Asians have a twofold higher risk of T2DM, while Black individuals have a 30% higher risk of T2DM. However, among the overweight or obese adults with newly diagnosed T2DM, mortality risk is highest in White, and lowest in Asian ethnic groups, and some of this differential risk is explained by clinical comorbidities and cardiovascular risk factors. This work underlines the influence of ethnicity on the risk of T2DM incidence but also ethnic disparity in risk of mortality associated with T2DM in obese or overweight adults. Knowledge and awareness of these ethnic variations are important for clinicians and policy-makers to aid priority-setting and ensure public health strategies are appropriately targeted. While findings underline the importance of enhanced screening for T2DM in Asians, they highlight concern for people from White backgrounds and emphasize the need for optimal disease and risk management appropriate to all communities with T2DM.

## Supplementary Information

Below is the link to the electronic supplementary material.

Supplementary file1 (DOCX 28 kb)

## Data Availability

The CPRD data analysed during this study are available from the Clinical Practice Research Datalink (CPRD) (enquiries@cprd.com) but restrictions apply to the availability of these data, which were used under license for the current study, and so are not publicly available. Data are, however, available from the authors upon reasonable request and with permission of the CPRD Independent Scientific Advisory Committee (ISAC) (enquiries@cprd.com).

## References

[1] World Health Organization (2016). Global report on diabetes.

[2] World Health Organization (2014). Global status report on noncommunicable diseases.

[3] Shai I, Jiang R, Manson JE, Stampfer MJ, Willett WC, Colditz GA (2006). Ethnicity, obesity, and risk of type 2 diabetes in women. Diabetes Care.

[4] Tillin T, Sattar N, Godsland IF, Hughes AD, Chaturvedi N, Forouhi NG (2015). Ethnicity-specific obesity cut-points in the development of type 2 diabetes—a prospective study including three ethnic groups in the United Kingdom. Diabet Med.

[5] Nwaneri C, Cooper H, Bowen-Jones D (2013). Mortality in type 2 diabetes mellitus: magnitude of the evidence from a systematic review and meta-analysis. Br J Diabetes Vasc Dis.

[6] Taylor KS, Heneghan CJ, Farmer AJ, Fuller AM, Adler AI, Aronson JK (2013). All-cause and cardiovascular mortality in middle-aged people with type 2 diabetes compared with people without diabetes in a large UK primary care database. Diabetes Care.

[7] Wright AK, Kontopantelis E, Emsley R, Buchan I, Sattar N, Rutter MK (2017). Life expectancy and cause-specific mortality in type 2 diabetes: a population-based cohort study quantifying relationships in ethnic subgroups. Diabetes Care.

[8] Chaturvedi N, Fuller JH (1996). Ethnic differences in mortality from cardiovascular disease in the UK: do they persist in people with diabetes?. J Epidemiol Community Health.

[9] Johns E, Sattar N (2017). Cardiovascular and mortality risks in migrant South Asians with type 2 diabetes: are we winning the battle?. Curr Diab Rep.

[10] Welcome to Clinical Practice Research Datalink (2020) https://www.cprd.com/home/

[11] Herrett E, Gallagher AM, Bhaskaran K, Forbes H, Mathur R, van Staa T (2015). Data resource profile: clinical practice research datalink (CPRD). Int J Epidemiol.

[12] Office for National Statistics (2018) Population of England and Wales by ethnicity. https://www.ethnicity-facts-figures.service.gov.uk/uk-population-by-ethnicity/national-and-regionalpopulations/population-of-england-and-wales/latest

[13] The English Index of Multiple Deprivation (IMD) (2015) In: Department for housing, communities and local government, editor. London 2015. https://www.gov.uk/government/statistics/english-indices-ofdeprivation-2015

[14] Hippisley-Cox J, Coupland C, Vinogradova Y, Robson J, Minhas R, Sheikh A (2008). Predicting cardiovascular risk in England and Wales: prospective derivation and validation of QRISK2. BMJ.

[15] Sherwani SI, Khan HA, Ekhzaimy A, Masood A, Sakharkar MK (2016). Significance of HbA1c test in diagnosis and prognosis of diabetic patients. Biomark Insights.

[16] Weir CB, Jan A (2021) BMI Classification percentile and cut off points. In: StatPearls. StatPearls Publishing. Treasure Island (FL) 31082114

[17] Pedersen AB, Mikkelsen EM, Cronin-Fenton D, Kristensen NR, Pham TM, Pedersen L (2017). Missing data and multiple imputation in clinical epidemiological research. Clin Epidemiol.

[18] Thompson D, Walhin JP, Batterham AM, Stokes KA, Cooper AR, Andrews RC (2014). Effect of diet or diet plus physical activity versus usual care on inflammatory markers in patients with newly diagnosed type 2 diabetes: the Early ACTivity in Diabetes (ACTID) randomized, controlled trial. J Am Heart Assoc.

[19] Wang J, Chen Y, Xu W, Lu N, Cao J, Yu S (2019). Effects of intensive blood pressure lowering on mortality and cardiovascular and renal outcomes in type 2 diabetic patients: a meta-analysis. PLoS ONE.

[20] Joseph JJ, Echouffo-Tcheugui JB, Golden SH, Chen H, Jenny NS, Carnethon MR (2016). Physical activity, sedentary behaviors and the incidence of type 2 diabetes mellitus: the Multi-Ethnic Study of Atherosclerosis (MESA). BMJ Open Diabetes Res Care.

[21] Wang M, Yi Y, Roebothan B, Colbourne J, Maddalena V, Wang PP (2016). Body mass index trajectories among middle-aged and elderly Canadians and associated health outcomes. J Environ Public Health.

[22] Wong ES, Wang BC, Alfonso-Cristancho R, Flum DR, Sullivan SD, Garrison LP (2012). BMI trajectories among the severely obese: results from an electronic medical record population. Obesity (Silver Spring).

[23] Iyen B, Weng S, Vinogradova Y, Akyea RK, Qureshi N, Kai J (2021). Long-term body mass index changes in overweight and obese adults and the risk of heart failure, cardiovascular disease and mortality: a cohort study of over 260,000 adults in the UK. BMC Public Health.

[24] Khan NA, Wang H, Anand S, Jin Y, Campbell NR, Pilote L (2011). Ethnicity and sex affect diabetes incidence and outcomes. Diabetes Care.

[25] Perry JR, Voight BF, Yengo L, Amin N, Dupuis J, Ganser M (2012). Stratifying type 2 diabetes cases by BMI identifies genetic risk variants in LAMA1 and enrichment for risk variants in lean compared to obese cases. PLoS Genet.

[26] Marshall JA, Hamman RF, Baxter J, Mayer EJ, Fulton DL, Orleans M (1993). Ethnic differences in risk factors associated with the prevalence of non-insulin-dependent diabetes mellitus: the San Luis valley diabetes study. Am J Epidemiol.

[27] Simmons D, Williams DRR, Powell MJ (1992). Prevalence of diabetes in different regional and religious South Asian communities in Coventry. Diabet Med.

[28] Oldroyd J, Banerjee M, Heald A, Cruickshank K (2005). Diabetes and ethnic minorities. Postgrad Med J.

[29] Shah BR, Victor JC, Chiu M, Tu JV, Anand SS, Austin PC (2013). Cardiovascular complications and mortality after diabetes diagnosis for South Asian and Chinese patients. Diabetes Care.

[30] Berrington-de-Gonzalez A, Hartge P, Cerhan JR, Flint AJ, Hannan L, MacInnis RJ (2010). Body-mass index and mortality among 1.46 million white adults. N Engl J Med.

[31] Calle EE, Thun MJ, Petrelli JM, Rodriguez C, Heath CW (1999). Body-mass index and mortality in a prospective cohort of US adults. N Engl J Med.

[32] Wen CP, David Cheng TY, Tsai SP, Chan HT, Hsu HL, Hsu CC (2009). Are Asians at greater mortality risks for being overweight than Caucasians? Redefining obesity for Asians. Public Health Nutr.

[33] WHO Expert (2004) Appropriate body-mass index for Asian populations and its implications for policy and intervention strategies. Lancet 363(9403):157–163 10.1016/S0140-6736(03)15268-3 14726171

