# Supplementary material for: Ethnic disparities in mortality among overweight or obese adults with newly diagnosed type 2 diabetes: a population-based cohort study
Source: J Endocrinol Invest. 2022 Jan 13;45(5):1011–20. doi: 10.1007/s40618-021-01736-9 (PMC8995280; doi:10.1007/s40618-021-01736-9)
Supplement: Supplementary file 1 — Supplementary file1 (DOCX 28 kb) [file 40618_2021_1736_MOESM1_ESM.docx]

**Online supplemental material**

**Supplemental table A**. HbA1c levels at T2DM diagnosis and one-year after diagnosis, in White, Asian and Black ethnic groups

| **Ethnic group** | **HbA1c (mmol/mol) at T2DM diagnosis (median(IQR))** | **HbA1c (mmol/mol) one year after T2DM diagnosis (median(IQR))** |
| --- | --- | --- |
| White | 54.10 (48.00 – 70.00) | 49.73 (44.00-58.00) |
| Asian | 56.28 (50.00 – 68.31) | 51.00 (46.45-59.56) |
| Black | 57.00 (50.00 – 72.00) | 50.82 (45.36-58.74) |

HbA1c at T2DM diagnosis, recorded in 76.7% of individuals (White 76.6%, Asian 75.4%, Black 79.2%)

HbA1c 1year after T2DM diagnosis recorded in 62% of individuals (White 62.3%, Asian 59.6%, Black 56.6%)

**Supplemental table B.** Unadjusted and adjusted hazards ratios for all-cause mortality in overweight and obese Black and Asian newly-diagnosed diabetics compared to Whites

(HbA1c at time of T2DM diagnosis included in adjusted model)

|  | White  N=27,356 | Asian  N=1,273 | Black  N=622 |
| --- | --- | --- | --- |
| **Overweight (BMI 25.0-29.9)**  Unadjusted Hazards Ratio (95% CI)  Adjusted Hazards ratio § | 1.00  1.00 | 0.27 (0.19-0.39)  0.51 (0.29-0.90) | 0.49 (0.28-0.84)  0.69 (0.33-1.46) |
| **Obesity class 1 (BMI 30-34.9)**  Unadjusted Hazards Ratio (95% CI)  Adjusted Hazards ratio § | 1.00  1.00 | 0.26 (0.18-0.38)  0.43 (0.23-0.78) | 0.40 (0.25-0.64)  0.77 (0.41-1.45) |
| **Obesity class 2 (BMI 35-39.9)**  Unadjusted Hazards Ratio (95% CI)  Adjusted Hazards ratio § | 1.00  1.00 | 0.44 (0.26-0.73)  0.72 (0.35-1.45) | 0.43 (0.21-0.85)  0.74 (0.28-2.00) |
| **Obesity class 3 (>40)**  Unadjusted Hazards Ratio (95% CI)  Adjusted Hazards ratio § | 1.00  1.00 | 0.61 (0.35-1.08)  0.95 (0.45-2.02) | 0.66 (0.36-1.19)  0.61 (0.29-1.29) |

§ Adjusted model has been adjusted for age of T2DM diagnosis, sex, baseline BMI, alcohol, smoking, deprivation, coronary heart disease, stroke, peripheral vascular disease, heart failure, statin use and HbA1c at diagnosis of T2DM

**Supplementary table C.** Hazards ratios for incidence of T2DM in obese or overweight Blacks and Asians, compared to those of White ethnicity (sensitivity analyses in only 139,520 individuals with linked secondary care data)

| **Ethnic group** | **Unadjusted Hazards ratio (95% CI)** | **Adjusted hazards ratio (95% CI)** | | |
| --- | --- | --- | --- | --- |
|  |  | **Model adjusted for age and sex** | **Model adjusted for baseline sociodemographic characteristics**** | **Model adjusted for baseline sociodemographic characteristics and clinical risk factors Ω** |
| White | 1.00 | 1.00 | 1.00 | 1.00 |
| Asian | 1.82 (1.72-1.92) | 2.13 (2.02-2.26) | 2.48 (2.35-2.63) | 2.05 (1.89-2.22) |
| Black | 1.03 (0.95-1.12) | 1.29 (1.19-1.40) | 1.23 (1.13-1.34) | 1.25 (1.12-1.39) |

**adjusted for age, sex, BMI, smoking, alcohol and deprivation

Ω adjusted for age, sex, BMI, smoking, alcohol, deprivation, hypertension, family history of T2DM, corticosteroid use and statin use

**Supplementary table D.** Hazards ratios for all-cause mortality in overweight and obese Black and Asians with newly diagnosed T2DM compared to Whites with newly diagnosed T2DM (sensitivity analyses in 27,137 individuals with linked secondary care data)

|  | White  N=24,800 | Asian  N=1,292 | Black  N=583 |
| --- | --- | --- | --- |
| **Overweight (BMI 25.0-29.9)**  Unadjusted Hazards Ratio (95% CI)  Adjusted Hazards ratio ¥ | 1.00  1.00 | 0.27 (0.19-0.39)  0.62 (0.40-0.97) | 0.49 (0.28-0.84)  0.64 (0.33-1.24) |
| **Obesity class 1 (BMI 30-34.9)**  Unadjusted Hazards Ratio (95% CI)  Adjusted Hazards ratio ¥ | 1.00  1.00 | 0.26 (0.18-0.38)  0.61 (0.40-0.95) | 0.40 (0.25-0.64)  0.77 (0.44-1.33) |
| **Obesity class 2 (BMI 35-39.9)**  Unadjusted Hazards Ratio (95% CI)  Adjusted Hazards ratio ¥ | 1.00  1.00 | 0.44 (0.26-0.73)  0.78 (0.45-1.36) | 0.43 (0.21-0.85)  0.64 (0.26-1.55) |
| **Obesity class 3 (BMI >40)**  Unadjusted Hazards Ratio (95% CI)  Adjusted Hazards ratio ¥ | 1.00  1.00 | 0.61 (0.35-1.08)  0.87 (0.49-1.55) | 0.66 (0.36-1.19)  0.73 (0.38-1.42) |

¥ adjusted for age of T2DM diagnosis, sex, baseline BMI, alcohol, smoking, deprivation, coronary heart disease, stroke, peripheral vascular disease, heart failure and statin use

**Supplemental table E. Mortality rates among obese or overweight adults without T2DM (n=155,362)**

|  | **White**  **N=147,195** | **Asian**  **N=4,372** | **Black**  **N=3,795** |
| --- | --- | --- | --- |
| **All-cause mortality** | | | |
| Number of deaths | 14,543 (9.88) | 145 (3.32) | 123 (3.24) |
| All-cause mortality rate (95% CI) per 1,000 p-years | 9.11 (8.96-9.26) | 3.73 (3.17-4.39) | 3.75 (3.15-4.48) |
| All-cause mortality rate (95% CI), by BMI  Overweight (25.0-29.9)  Obesity class 1 (30-34.9)  Obesity class 2 (35-39.9)  Obesity class 3 (>40) | 8.82 (8.58-9.07)  9.26 (9.02-9.52)  8.56 (8.23-8.91)  10.52 (10.03-11.03) | 3.29 (2.53-4.29)  3.50 (2.67-4.59)  4.45 (2.96-6.70)  6.93 (4.18-11.49) | 2.89 (1.98-4.21)  3.84 (2.89-5.10)  3.79 (2.58-5.56)  5.41 (3.56-8.22) |
| **CVD-related mortality** | | | |
| Number of CVD deaths | 2598 | 31 | 21 |
| CVD mortality rate (95% CI) per 1,000 p-years | 1.63 (1.57-1.69) | 0.80 (0.56-1.13) | 0.64 (0.42-0.98) |

**Supplemental table F. Hazards ratios for all-cause mortality in the general population of non-T2DM overweight and obese Black and Asian individuals compared to Whites**

|  | White  N=147,195 | Asian  N=4,372 | Black  N=3,795 |
| --- | --- | --- | --- |
| **Overweight (BMI 25.0-29.9)**  Unadjusted Hazards Ratio (95% CI)  Adjusted Hazards ratio ¥ | 1.00  1.00 | 0.45 (0.34-0.58)  0.99 (0.72-1.36) | 0.37 (0.25-0.54)  0.87 (0.58-1.32) |
| **Obesity class 1 (BMI 30-34.9)**  Unadjusted Hazards Ratio (95% CI)  Adjusted Hazards ratio ¥ | 1.00  1.00 | 0.45 (0.34-0.59)  1.11 (0.82-1.51) | 0.48 (0.36-0.64)  1.11 (0.81-1.51) |
| **Obesity class 2 (BMI 35-39.9)**  Unadjusted Hazards Ratio (95% CI)  Adjusted Hazards ratio ¥ | 1.00  1.00 | 0.59 (0.39-0.89)  1.10 (0.71-1.72) | 0.53 (0.36-0.78)  0.84 (0.54-1.29) |
| **Obesity class 3 (BMI >40)**  Unadjusted Hazards Ratio (95% CI)  Adjusted Hazards ratio ¥ | 1.00  1.00 | 0.71 (0.43-1.18)  1.13 (0.64-1.99) | 0.60 (0.39-0.91)  0.86 (0.55-1.35) |

¥ adjusted for baseline age, sex, alcohol, smoking, deprivation, coronary heart disease, stroke, peripheral vascular disease, heart failure and statin use

**List of CPRD Medical codes for Type 2 Diabetes (Non-Insulin Dependent Diabetes Mellitus)**

| Medical code | Read code | Read term |
| --- | --- | --- |
| 64668 | C10FJ11 | Insulin treated Type II diabetes mellitus |
| 106061 | C10FP11 | Type II diabetes mellitus with ketoacidotic coma |
| 36633 | C109K00 | Hyperosmolar non-ketotic state in type 2 diabetes mellitus |
| 17262 | C109600 | Non-insulin-dependent diabetes mellitus with retinopathy |
| 62674 | C10FA00 | Type 2 diabetes mellitus with mononeuropathy |
| 61071 | C109D12 | Type 2 diabetes mellitus with hypoglycaemic coma |
| 26054 | C10FL00 | Type 2 diabetes mellitus with persistent proteinuria |
| 37806 | C10FF00 | Type 2 diabetes mellitus with peripheral angiopathy |
| 108005 | C109312 | Type 2 diabetes mellitus with multiple complications |
| 98616 | C10F211 | Type II diabetes mellitus with neurological complications |
| 40401 | C109500 | Non-insulin dependent diabetes mellitus with gangrene |
| 49074 | C10F400 | Type 2 diabetes mellitus with ulcer |
| 1407 | C10FJ00 | Insulin treated Type 2 diabetes mellitus |
| 18278 | C109J00 | Insulin treated Type 2 diabetes mellitus |
| 50527 | C10FB11 | Type II diabetes mellitus with polyneuropathy |
| 107701 | C10FK11 | Hyperosmolar non-ketotic state in type II diabetes mellitus |
| 758 | C10F.00 | Type 2 diabetes mellitus |
| 36695 | C10D.00 | Diabetes mellitus autosomal dominant type 2 |
| 24693 | C109G00 | Non-insulin dependent diabetes mellitus with arthropathy |
| 51756 | C10FP00 | Type 2 diabetes mellitus with ketoacidotic coma |
| 18390 | C10FM00 | Type 2 diabetes mellitus with persistent microalbuminuria |
| 58604 | C109611 | Type II diabetes mellitus with retinopathy |
| 42762 | C109612 | Type 2 diabetes mellitus with retinopathy |
| 95351 | C10FA11 | Type II diabetes mellitus with mononeuropathy |
| 56268 | C109D11 | Type II diabetes mellitus with hypoglycaemic coma |
| 59725 | C109111 | Type II diabetes mellitus with ophthalmic complications |
| 34268 | C10F200 | Type 2 diabetes mellitus with neurological complications |
| 34912 | C109400 | Non-insulin dependent diabetes mellitus with ulcer |
| 25591 | C10FQ00 | Type 2 diabetes mellitus with exudative maculopathy |
| 47954 | C10F900 | Type 2 diabetes mellitus without complication |
| 98723 | C10FD11 | Type II diabetes mellitus with hypoglycaemic coma |
| 59253 | C10FG00 | Type 2 diabetes mellitus with arthropathy |
| 34450 | C10FK00 | Hyperosmolar non-ketotic state in type 2 diabetes mellitus |
| 18264 | C109J12 | Insulin treated Type II diabetes mellitus |
| 37648 | C109J11 | Insulin treated non-insulin dependent diabetes mellitus |
| 50429 | C109100 | Non-insulin-dependent diabetes mellitus with ophthalm comps |
| 18425 | C10FB00 | Type 2 diabetes mellitus with polyneuropathy |
| 48192 | C109E11 | Type II diabetes mellitus with diabetic cataract |
| 70316 | C109112 | Type 2 diabetes mellitus with ophthalmic complications |
| 57278 | C10F011 | Type II diabetes mellitus with renal complications |
| 64571 | C109C11 | Type II diabetes mellitus with nephropathy |
| 85991 | C10FM11 | Type II diabetes mellitus with persistent microalbuminuria |
| 24836 | C109C12 | Type 2 diabetes mellitus with nephropathy |
| 50609 | L180600 | Pre-existing diabetes mellitus, non-insulin-dependent |
| 55842 | C109200 | Non-insulin-dependent diabetes mellitus with neuro comps |
| 65267 | C10F300 | Type 2 diabetes mellitus with multiple complications |
| 62146 | C109300 | Non-insulin-dependent diabetes mellitus with multiple comps |
| 55075 | C109411 | Type II diabetes mellitus with ulcer |
| 53392 | C10F911 | Type II diabetes mellitus without complication |
| 46917 | C10FD00 | Type 2 diabetes mellitus with hypoglycaemic coma |
| 12640 | C10FC00 | Type 2 diabetes mellitus with nephropathy |
| 44779 | C109E12 | Type 2 diabetes mellitus with diabetic cataract |
| 103902 | C10FG11 | Type II diabetes mellitus with arthropathy |
| 54899 | C109F11 | Type II diabetes mellitus with peripheral angiopathy |
| 60699 | C109F12 | Type 2 diabetes mellitus with peripheral angiopathy |
| 105784 | C109912 | Type 2 diabetes mellitus without complication |
| 109103 | C109911 | Type II diabetes mellitus without complication |
| 18777 | C10F000 | Type 2 diabetes mellitus with renal complications |
| 59365 | C109C00 | Non-insulin dependent diabetes mellitus with nephropathy |
| 12736 | C10F500 | Type 2 diabetes mellitus with gangrene |
| 66965 | C109H12 | Type 2 diabetes mellitus with neuropathic arthropathy |
| 47816 | C109H11 | Type II diabetes mellitus with neuropathic arthropathy |
| 67905 | C109211 | Type II diabetes mellitus with neurological complications |
| 45919 | C109212 | Type 2 diabetes mellitus with neurological complications |
| 43227 | C10F311 | Type II diabetes mellitus with multiple complications |
| 49655 | C10F611 | Type II diabetes mellitus with retinopathy |
| 106528 | C10FN11 | Type II diabetes mellitus with ketoacidosis |
| 44982 | C10FE00 | Type 2 diabetes mellitus with diabetic cataract |
| 4513 | C109.00 | Non-insulin dependent diabetes mellitus |
| 47321 | C10F100 | Type 2 diabetes mellitus with ophthalmic complications |
| 47315 | C10F711 | Type II diabetes mellitus - poor control |
| 24458 | C109711 | Type II diabetes mellitus - poor control |
| 45913 | C109712 | Type 2 diabetes mellitus - poor control |
| 104323 | C10F511 | Type II diabetes mellitus with gangrene |
| 50813 | C109A11 | Type II diabetes mellitus with mononeuropathy |
| 102201 | C10FC11 | Type II diabetes mellitus with nephropathy |
| 52303 | C109000 | Non-insulin-dependent diabetes mellitus with renal comps |
| 29979 | C109900 | Non-insulin-dependent diabetes mellitus without complication |
| 45467 | C109B00 | Non-insulin dependent diabetes mellitus with polyneuropathy |
| 18219 | C109.13 | Type II diabetes mellitus |
| 46150 | C109512 | Type 2 diabetes mellitus with gangrene |
| 18496 | C10F600 | Type 2 diabetes mellitus with retinopathy |
| 50225 | C109011 | Type II diabetes mellitus with renal complications |
| 32627 | C10FN00 | Type 2 diabetes mellitus with ketoacidosis |
| 93727 | C10FE11 | Type II diabetes mellitus with diabetic cataract |
| 5884 | C109.11 | NIDDM - Non-insulin dependent diabetes mellitus |
| 17859 | C109.12 | Type 2 diabetes mellitus |
| 100964 | C10F111 | Type II diabetes mellitus with ophthalmic complications |
| 43785 | C109D00 | Non-insulin dependent diabetes mellitus with hypoglyca coma |
| 60796 | C10FL11 | Type II diabetes mellitus with persistent proteinuria |
| 8403 | C109700 | Non-insulin dependent diabetes mellitus - poor control |
| 72320 | C109A00 | Non-insulin dependent diabetes mellitus with mononeuropathy |
| 109197 | C10FH11 | Type II diabetes mellitus with neuropathic arthropathy |
| 63690 | C10FR00 | Type 2 diabetes mellitus with gastroparesis |
| 18209 | C109012 | Type 2 diabetes mellitus with renal complications |
| 506 | C100112 | Non-insulin dependent diabetes mellitus |
| 47409 | C109B11 | Type II diabetes mellitus with polyneuropathy |
| 109865 | C109B12 | Type 2 diabetes mellitus with polyneuropathy |
| 62107 | C109511 | Type II diabetes mellitus with gangrene |
| 91646 | C10F411 | Type II diabetes mellitus with ulcer |
| 25627 | C10F700 | Type 2 diabetes mellitus - poor control |
| 18143 | C109G11 | Type II diabetes mellitus with arthropathy |
| 35385 | C10FH00 | Type 2 diabetes mellitus with neuropathic arthropathy |
| 104639 | C10FF11 | Type II diabetes mellitus with peripheral angiopathy |
| 22884 | C10F.11 | Type II diabetes mellitus |
| 49869 | C109G12 | Type 2 diabetes mellitus with arthropathy |
